# Coronary Atherosclerosis T1-weighed Characterization with integrated anatomical reference (CATCH)

**DOI:** 10.1186/1532-429X-18-S1-O22

**Published:** 2016-01-27

**Authors:** Yibin Xie, Jianing Pang, Young Jin Kim, Qi Yang, Jung-Sun Kim, Christopher T Nguyen, Byoung Wook Choi, Zhaoyang Fan, Daniel S Berman, Hyuk-Jae Chang, Debiao Li

**Affiliations:** 1 grid.50956.3f0000000121529905Cedars-Sinai Medical Center, Los Angeles, CA USA; 2 grid.15444.300000000404705454Yonsei University College of Medicine, Seoul, Korea (the Republic of)

## Background

The detection of high-risk coronary atherosclerotic lesions before severe plaque complications is the "holy grail" in cardiology. Recently T1-weighted (T1w) MRI with [2] or without [3] contrast enhancement (CE) has been used for characterizing coronary plaques showing promising prognostic value [4]. However the drawbacks of current protocols based on conventional Cartesian acquisition and navigator gating hinder the clinical application of this technique: a) coverage is limited to proximal coronary segments; b) spatial resolution is low and often anisotropic; c) because normal tissue in T1w images is highly suppressed, a separate MRA acquisition is needed to provide anatomical reference. **The purpose of this work is to develop a highly accelerated MR technique for coronary plaque characterization with 1) whole-heart coverage, 2) fine isotropic spatial resolution, and 3) simultaneously acquired bright-blood anatomical reference.**

## Methods

**Figure 1:**
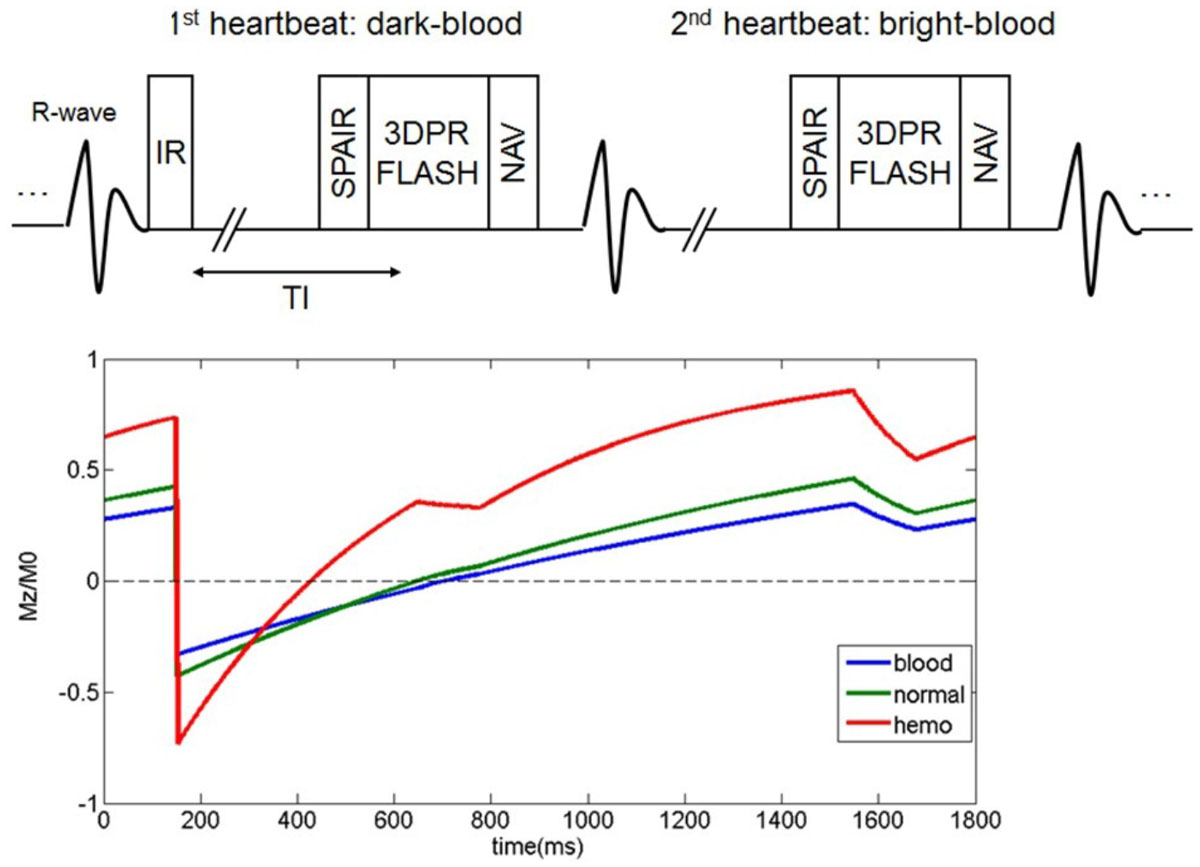
**Upper panel: Sequence diagram of CATCH**. Lower panel: Simulated steady-state signal behavior of blood, normal vessel wall, and intra-plaque hemorrhage.

CATCH consists of ECG-gated, inversion recovery (IR) prepared spoiled gradient echo sequence with golden angle 3D radial trajectory to acquire dark-blood T1w images and bright-blood reference images in an interleaved fashion (Fig. 1). Retrospective motion correction with 100% respiratory gating efficiency was performed as described previously [5]. Healthy volunteers (n = 12) and CAD patients with stable and unstable angina (n = 26) were scanned on a 3T scanner (Siemens Magnetom Trio) before and after CE. Scan parameters: whole-heart 3D slab with FOV = 330^3^ mm^3^; spatial resolution = 1.1^3^ mm^3^; TR/TE = 4.6/2.3 ms; number of radial projections = 8500; scan time = ~10 minutes depending on heart rate. After completing MRI, 21 CAD patients further underwent interventional X-ray angiography (XA) and intracoronary optical coherence tomography (OCT) for coronary plaque evaluation. OCT images were graded for high-risk coronary plaque features (lipid-richness, macrophages, microvessels, cholesterol crystals) by two experienced cardiologists without the knowledge of MR results.

## Results

**Figure 2:**
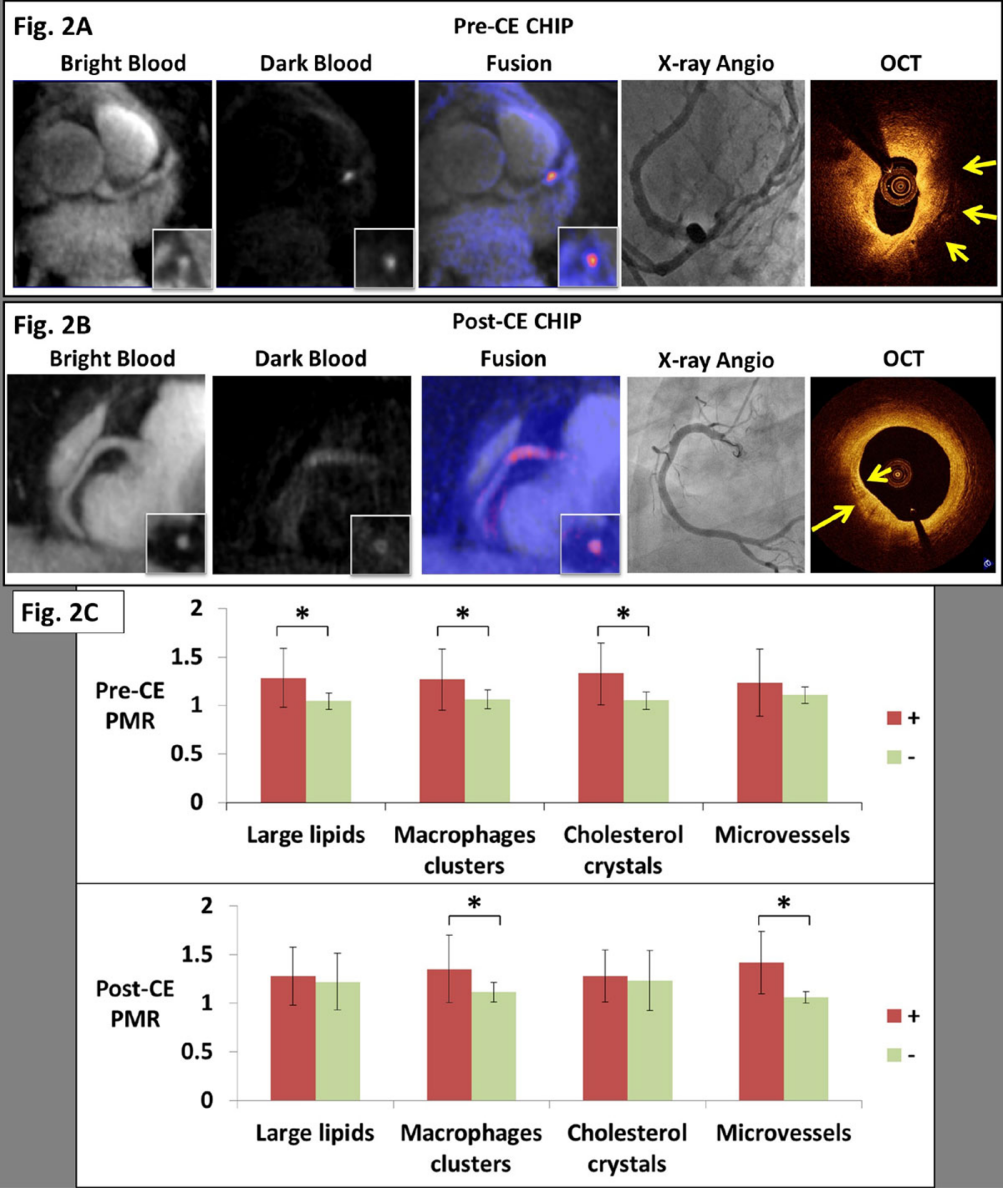
**A: An example of pre-CE CHIP was found at middle LAD as localized on the bright-blood images**. XA showed significant stenosis (70%) at that location. OCT showed large signal-poor area suggestive of possible lipid core and/or intra-plaque hemorrhage (yellow arrow). **B**: An example of post-CE CHIP with diffuse wall enhancement at proximal RCA as localized on the bright-blood images. XA showed only mild stenosis (30%) at that location. OCT showed strong multi-focal back reflections and signal heterogeneity within the overlaying tissue suggestive of high macrophage density (yellow arrows). **C**: Coronary plaques with high-risk features as classified by OCT tended to be hyper-intensive on CATCH images. Star signs (*) denote statistical significance (p < 0.05). Positive sign (+) and negative sign (-) denote lesion groups with corresponding OCT grading. Plaque hyper-intensity is presented in terms of plaque to myocardium ratio (PMR) as described previously [4].

All 38 subjects successful completed the pre-CE exams. All 12 healthy volunteers and 23 eligible patients also completed the post-CE exams. None of the healthy subjects showed coronary hyper-intensive plaques (CHIPs) in either pre-CE or post-CE T1w MRI. In total 3 patients showed CHIPs on pre-CE exams and 4 patients showed CHIPs on post-CE exams, respectively. Fig. 2A and Fig. 2B are two representative patient cases with a pre-CE CHIP and a post-CE CHIP, respectively, with corresponding imaging evidences from other modalities. Fig. 2C is the lesion-based statistics showing elevated plaque hyper-intensity in the advanced lesions as classified by OCT.

## Conclusions

The proposed MR technique of accelerated T1w whole heart coronary plaque characterization with simultaneously acquired anatomical reference was feasible. Coronary plaque hyper-intensity showed positive association with certain high-risk plaque features on OCT.

